# The agreement between the referrer, practitioner and research diagnosis of autistic spectrum conditions among children attending child and adolescent mental health services

**DOI:** 10.1007/s00787-019-01290-z

**Published:** 2019-02-18

**Authors:** Tamsin Ford, Ralphy Kenchington, Shelley Norman, John Hancock, Alex Smalley, William Henley, Ginny Russell, Jennie Hayes, Stuart Logan

**Affiliations:** 1 University of Exeter Medical School, St Luke’s Campus, 2.03 College House, College Road, Devon, Exeter, EX1 2LU England; 2 Psychology Department, University of Exeter, Exeter, England; 3 Centre for Environment and Human Health, University of Exeter, Exeter, England

**Keywords:** Autistic spectrum conditions, Diagnostic agreement, Diagnostic stability, CAMHS

## Abstract

We aimed to explore the levels of agreement about the diagnoses of Autistic Spectrum Conditions between the referrer, CAMHS practitioner and a research diagnosis, as well as the stability of the practitioner’s diagnosis over time in a secondary analysis of data from 302 children attending two Child and Adolescent Mental Health Services over two years. Kappa coefficient was used to assess the agreement between the referrer and research diagnosis. Kendall’s tau *b* coefficient was used to assess the agreement between the practitioner and the research diagnosis assigned using the Development and Well-Being Assessment, as well as the agreement between the referrer’s indication of presenting problems and the practitioner diagnosis. Diagnostic stability was explored in children with and without a research diagnosis of Autistic Spectrum Condition. There was a moderate level of agreement between the referrer and research diagnosis (Kappa = 0.51) and between practitioner’s and research diagnosis (Kendall’s tau = 0.60) at baseline, which reduced over the subsequent two years. Agreement between the referrer and practitioner’s diagnosis at baseline was fair (Kendall’s tau = 0.36).The greatest diagnostic instability occurred among children who practitioners considered to have possible Autistic Spectrum Conditions but who did not meet research diagnostic criteria. Further studies could explore the approaches used by practitioners to reach diagnoses and the impact these may have on diagnostic stability in Autistic Spectrum Conditions. Standardised assessment using a clinically rated diagnostic framework has a potential role as an adjunct to standard clinical care and might be particularly useful where practitioners are uncertain.

## Introduction

The issue of accurate and timely diagnosis for children with Autistic Spectrum Conditions (ASCs) is important because specialist services and interventions can often only be accessed after a formal diagnosis [1, 2]. ASCs are neurodevelopmental disorders characterised by persistent difficulties with social communication and social interaction combined with repetitive patterns of behaviours, activities or interests (including sensory behaviour) that impair daily functioning [3]. Many children with ASCs experience persistent impairment into adulthood, and some remain highly dependent on others [4]. ASCs have a prevalence of approximately 0.6–15 per thousand among school-aged children and are more frequently reported in boys (male to female ratio = 4:1) [5, 6]. The identification of ASCs may be increasing [7], and although changes to the Special Educational Needs System make comparison difficult, the number of Education and Health Care Plans that list ASCs as a primary difficulty has increased from 19% in 2010 to just over a quarter in 2017 [8].

Reports that intensive early behavioural interventions for children with ASCs may lead to some progress for some children with ASCs in some domains, such as cognitive performance, language skills and adaptive behaviours [9], would suggest that prompt identification is important. The National Autism Plan for Children recommended that the time from referral for specialist assessment to a diagnosis/feedback should not exceed 17 weeks [10], while the National Institute for Clinical Excellence guidance suggested that diagnostic assessments should commence within three months of referral [11]. In contrast, parents frequently report delays that ranged between 18 months and 11 years in obtaining a diagnosis of ASCs, even when their child is attending Child and Adolescent Mental Health Services (CAMHS) [12, 13]. Waiting times for a diagnosis have long been a concern [14], and may be increasing in the UK [15], with over half of child development teams in the UK reporting that they were unable to provide a defined timescale for the completion of ASC diagnostic assessment [16]. Studies suggest that as many as half of parents accessing assessments are dissatisfied with the referral and assessment process [17, 18]. Many report seeing multiple different professionals prior to finally obtaining a diagnosis for their child [19], which results in long, stressful waiting times and delays in access to services, support and interventions [19–22].

There is no one standardised ‘gold standard’ assessment tool that is consistently used to diagnose ASCs, although a wide variety of standardised interviews, screens and observational measures are applied in practise. Arguably, the Autism Diagnostic Interview combined with the Autism Diagnostic Observation Schedule is considered to be a reference standard to aspire to in research and clinical practise [23]. To access assessment, a child needs to be referred to a specialist service, which is frequently a CAMHS or community paediatrics. Specialist services differ in who they will accept referrals from, but common sources of referrals include general practitioners, schools and sometimes parents themselves. The process of referral commonly involves sending a letter or completing a form that summarises the child’s difficulties or presenting problems, and supplies additional information about the child and their family. Assessment often involves clinical interview (s), with or without the application of a standardised diagnostic assessment, collateral history from key informants and/or observation and/or the use of rating scales. The NICE guidelines [11] recommend that practitioners do not rely on a single autism-specific diagnostic tool. The variation in approach seen across different services might underpin some of the delays reported by parents in accessing a diagnosis and service [24, 25]. The current study aimed to explore the level of agreement of a standardised diagnosis of ASC among primary school children attending two CAMHS with the opinions of referrers and with practitioners in relation to the severity of children’s difficulties, as well as to document the certainty and stability of the practitioners’ diagnoses and the time between referral and definite diagnosis of ASC by a practitioner.

## Methodology

### Design

This was a secondary analysis of data from a cohort of children aged 5–11 years attending two CAMHS collected between 2006 and 2008 [26]. The original study had NHS Research ethics committee approval from the Joint South London and Maudsley and Institute of Psychiatry Joint Research Ethics Committee to explore the agreement between research and practitioner diagnosis, and was conducted in compliance with the protocol, good clinical practise and regulatory requirements.

## Clinical setting

CAMHS are based within the UK’s National Health Service. Four tiers of provision are widely adopted in service provision. The primary study was set within two general CAMHS in an urban area, with populations which were broadly representative of the British population. CAMHS A was the only service in Area A that assessed children and young people with psychiatric disorder. This study was based in the Children’s Team (Tier 3/secondary care), which provided multidisciplinary treatments to children up to the age of 16 and the Early Interventions Team (Tier 2/primary care) for children who have less severe problems or were less likely to engage with the traditional CAMHS service. CAMHS B consisted of a single multidisciplinary team with specialist sub-teams for ADHD, adolescents, and looked after children (Tier 3). Tier 2 services in CAMHS B were provided by a separate team, while neither CAMHS A nor B provided highly specialised (Tier 4/tertiary care) services.

### Participants

The participants were recruited from 861 consecutive referrals accepted on the CAMHS waiting lists during the recruitment period (April 2006–March 2008 CAMHS A; March 2007–July 2008 CAMHS B). The inclusion criteria included age between 5 and 10 years 9 months at the time that they were accepted onto the waiting list to ensure a relatively homogenous sample of primary school age children. There were three exclusion criteria: first, if the child was looked after by their local authority, because of the difficulty of changes in parental responsibility during the course of the study and because of the difficulty in finding informants that knew the child well enough to complete the study’s measures reliably. Parents with insufficient English to complete the questionnaires were also excluded. Finally, emergency and urgent paediatric liaison referrals were excluded because of the difficulty in gaining consent and completing the baseline assessment between referral and first assessment.

### Measures

#### Research diagnosis of ASC using the Development and Well-Being Assessment (DAWBA) [27]

The Development and Well-Being Assessment (DAWBA) is a standardised diagnostic assessment tool that combines highly structured questions that directly relate to DSM [3, 28] and ICD 10 [29] research diagnostic criteria, with semi-structured comments about any reported difficulties. In the current study, parents and if they agreed, teachers, were invited to complete the DAWBA as the children were too young to complete it reliably themselves. Answers to the structured questions and the qualitative data from informants can then be combined via computer algorithm to produce a probability of common childhood psychiatric diagnoses, including ASC. It is then possible to review the responses from all informants to both structured and semi-structured questions to assign diagnoses. For the purpose of the original study, all 302 cases were clinically rated according to ICD 10 by TF, blind to what practitioners had reported [29]. Clinical rating allows the clinician to moderate diagnoses according to conflicting information from different informants in the way that they would in clinical practise, to detect when informants have misunderstood the question and to assign “not otherwise specified” diagnoses for children whose difficulties are clinically significant but do not meet diagnostic criteria.

The DAWBA provided excellent discrimination between community and clinical samples for a range of common childhood psychiatric disorders [27]. Within the community sample, children with DAWBA diagnoses differed markedly from those without a disorder in both external characteristics and prognosis as would be predicted from aetiological and epidemiological research. There were also high levels of agreement between the DAWBA and case notes among the clinical sample about emotional, behavioural and hyperkinetic disorders (Kendall’s tau *b* = 0.47–0.70). There is no test–retest reliability of the DAWBA as attenuation for such an in-depth assessment would be so great as to render any such assessment invalid [27]. The DAWBA has been shown to have high levels of sensitivity (0.88) and specificity (0.85) in detecting ASC in a population-based twin study, when it correlated highly (*ρ* =0.82, *p* < 0.001) with the best estimate research diagnosis (revised Autism Diagnostic Interview combined with the Autism Diagnostic Observation Schedule) [23].

### Presenting problems on referral

Presenting problems for each child were extracted from referral letters by research workers at the time of recruitment and then classified into independent categories coded present or absent, including ASC. Multiple problems could be endorsed for a single child if necessary. The reliability of these categories was established by comparing the initial categorisation with the application of the same categories by an experienced child and adolescent psychiatrist who independently classified the presenting problems across the whole sample using the same scheme. There was 91% agreement about whether referral letters suggested that a child might have an ASC (Kappa = 0.76).

### Practitioner diagnosis of ASC

It was assumed that practitioners were working and making diagnoses within currently accepted clinical guidelines for their profession. This would, therefore, usually comprise of a clinical assessment. The use of standardised assessments such as ADOS/ADI is not part of routine practise and not recommended to be used in isolation (although these may have been used as part of an assessment by some specialist teams). Data on practitioner diagnosis of ASC and other disorders were collected via a brief questionnaire at baseline and at each follow-up while the child continued to attend the clinic. This questionnaire included a list of psychiatric disorders assessed by the DAWBA, supplemented by difficulties that the participating CAMHS practitioners thought should be included. It covered separation anxiety, specific phobia, social phobia, generalised anxiety, obsessive compulsive disorder, post-traumatic stress disorder, other anxiety, depression, oppositional-defiant disorder, conduct disorder, attention deficit hyperactivity disorder, autistic spectrum disorders, eating disorders, selective mutism, tic disorders and other difficulties. Practitioners could respond “no”, “possible” or “definite” for each diagnosis and were able to endorse as many disorders as they felt were applicable in each case. The practitioner report was completed by the case manager for that child at that particular time point, which varied for some children over time.

The study, therefore, had three measures of the children’s difficulties which are summarised in Table 1; referral, research, and practitioner).

**Table 1:** Explanation of the different diagnoses of Autistic Spectrum Conditions and where the data were obtained from

Term used	Method of obtaining data	Method of checking reliability	Possible outcomes
Referral or presenting problems	Extracted from referral letters (e.g. from general practitioners, schools) by researchers at baseline	Compared to results of independent classification by experienced child and adolescent psychiatrist	Suggests ASC Does not suggest ASC
Practitioner diagnosis	Reported by child’s case manager at each time point provided the child was still attending the clinic (e.g. CAMHS psychiatrist, clinical psychologist)		ASC according to practitioner: Definite Possible No
Research diagnosis	DAWBA completed by parents and some teachers Results clinically rated according to ICD-10 by independent experienced clinician at baseline	Previous literature on test reliability	Research diagnosis of ASC No research diagnosis of ASC

### Parental report of child’s psychopathology

Parents also completed the Strengths and Difficulties Questionnaire (SDQ) at baseline. In this validated questionnaire [30], 25 items are divided between five subscales, generating scores for conduct problems, hyperactivity–inattention, emotional symptoms, peer relationships and prosocial behaviours. All but the last subscale are summed to generate a total difficulties score. Items are phrased as either a positive or negative statement and informants select between the following responses; “not true”, “somewhat true” or “certainly true”, scored 0, 1, 2 or reversed with positive statements. Thus, a high score indicates greater difficulty, with the exception of the prosocial scale in which higher scores indicate better functioning. The SDQ impact supplement generates an impact score based on ratings of child distress and the impact of difficulties on home life, friendship, classroom learning, and leisure activities. An additional question asks about the burden of the psychopathology to the informant (rated ‘not at all’, ‘only a little’, ‘quite a lot’ and ‘a great deal’).

### Procedure

Once a referral was accepted into the waiting list, the family was contacted about the research. If the parents consented, they were asked to complete the DAWBA prior to their first appointment at CAMHS and if they agreed, a shortened version of the DAWBA was sent to the child’s teacher. The DAWBA includes the SDQ at the beginning of the assessment. Two hundred and seventy-nine parents consented to the completion of a teacher DAWBA (92% of the final sample) and of these 206 (74%) were completed.

As part of a wider randomised control trial (RCT) nested into this cohort study [31], the unrated DAWBA assessments were disclosed to approximately half of the practitioners (43%) before they conducted their first assessment with the child. The DAWBA RCT found no statistically significant effect of disclosure of the DAWBA upon diagnostic agreement between practitioners and the DAWBA, but the levels of agreement found in this secondary analysis will be stratified by DAWBA disclosure to account for the increased level of information available to some practitioners about some children.

After the first assessment at the clinic, the practitioner was asked to provide their assessment of the child’s difficulties. Over the next 2 years practitioners reported their diagnoses (referred to as practitioner diagnoses below) at 6-month intervals.

**Fig. 1:**
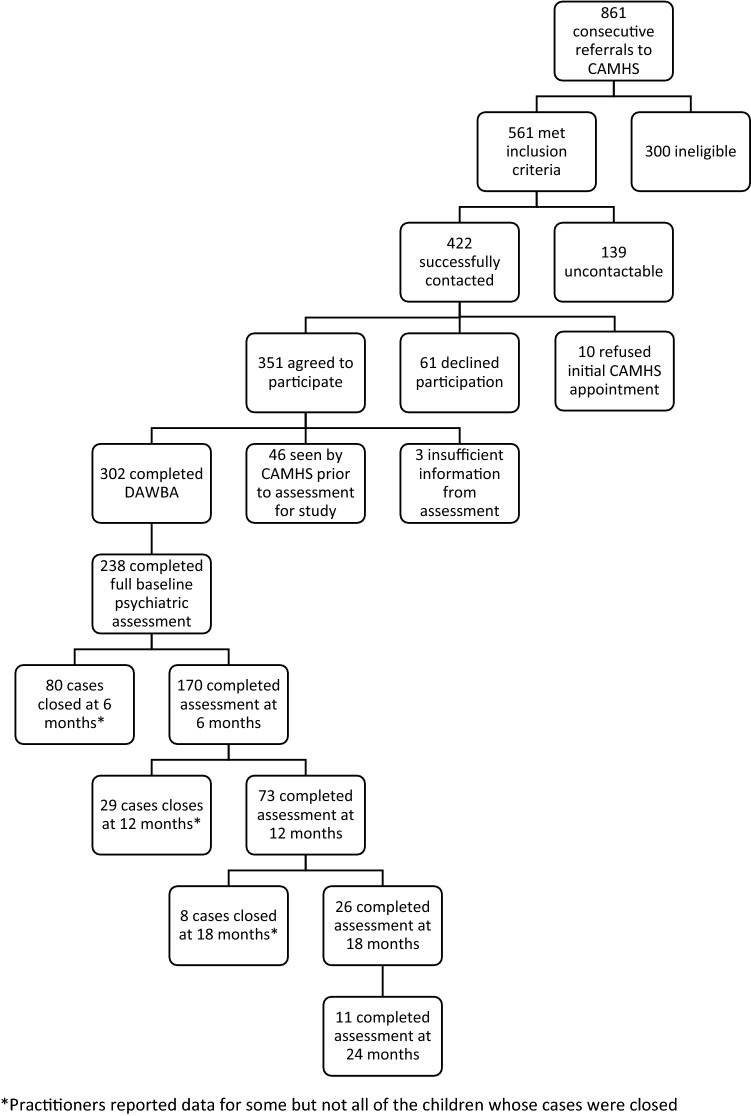
Flow diagram of participants

The flow of participants is depicted in Fig. 1. Of the 861 consecutive referrals, 561 children (65%) met the inclusion criteria; nearly two-thirds of those eligible for inclusion (*n* = 351 or 62%) agreed to participate. Forty-six of these children were seen by the clinic before the research team could assess them, either because a crisis resulted in the family’s appointment being brought forward, which also meant that they were no longer eligible (*n* = 22 or 48%) or when a new members of staff with an empty diary took multiple cases off the waiting list and omitted to inform the research team (*n* = 24 or 52%). Ten families refused the offer of an appointment at CAMHS when it was first sent, taking the ineligible total to 356. The researchers were unable to contact 139 eligible families, while 61 declined participation. Upon data cleaning, three children had too little data from the parental assessment completed to be included, which left the final sample of 302 children with research diagnoses (55% of those eligible for inclusion and 72% of those whom the researchers managed to contact). Analysis of practitioner diagnoses begins at Time 1 with 238 participants because some practitioners failed to provide full data about assessment.

### Analysis

The analysis was conducted in SPSS 20.0. The DAWBA rated by TF (henceforth, referred to as the research diagnosis) was taken to be the reference standard, although with the proviso that a true gold standard comparison is conceptually problematic for clinical psychiatric diagnoses.

The number of children with data returned at each time point decreased, which was an inevitable consequence of children being discharged from or leaving the service as well as loss to follow-up. The numbers by the fifth time point were too small for statistical analysis (*n* = 11, *n* = 5 with ASC according to the DAWBA rated by TF); therefore, we present analysis on data from baseline to 18-month follow-up, and descriptive data only for the last follow-up for those who may be interested.

The proportion of children with a practitioner diagnosis of ASC at any time point was cross tabulated with the research diagnosis, as were the presenting problems. Chance-corrected agreement was assessed using the Kappa statistic, which was interpreted utilising the levels assigned by Landis and Koch [32]: Kappa of 0.21–0.40 = fair, 0.41–0.60 = moderate and 0.61–0.80 = substantial agreement. Practitioner diagnoses at each time point were compared to the research diagnoses as well as to the referrers’ queries about ASC in the presenting problems using Kendall’s tau *B* as Kappa could not be calculated for a two by three cross tabulation.

Those with a research diagnosis of ASC (*n* = 41) and those without (*n* = 201) were explored in terms of diagnostic stability stratified by Time 1 practitioner diagnosis (definite, possible or no ASC) to explore the stability of practitioner diagnoses. Statistical analysis of these would be of limited value due to the high rate of attrition, but figures have been included for information. Practitioners could only provide diagnoses for children who were still attending the clinic.

Finally, we explored the baseline parent-reported peer relationship and prosocial skills of those with or without a research diagnosis of ASC. As practitioners reported very few children to have a definite ASC diagnoses who were not also assigned a research diagnosis (*n* = 3), we could not analyse them as a separate group and these three children were combined with those with a possible practitioner diagnosis (Table 2).

**Table 2:** Agreement between referral and research diagnosis with Kappa coefficient score

Referral	Research diagnosis according to DAWBA, *N *(%)		Kappa
	Research diagnosis of ASC	No research diagnosis of ASC	Value
Suggests ASC	29 (49%)	30 (51%)	0.51 (*p* = 0.00)
Does not suggest ASC	12 (5%)	231 (95%)	

## Results

Of the 302 children recruited, 41 (13.5%) had a research ASC diagnosis. For 21 of these (51%), the DAWBA results were made available to the assessing practitioners. The levels of agreement shown in Table 3 were also calculated, stratified by DAWBA disclosure (Tables 4 and 5), which had little impact on levels of agreement and no statistically significant effect. Thus, further analysis was conducted without stratifying by trial arm.

**Table 3:** Agreement between practitioner and clinically rated research diagnosis across four time points with Kendall’s tau b coefficient score

Time point	Number of children with practitioner data	Number of children with a research diagnosis of ASC	Type of diagnosis	ASC according to practitioner *N* (%)			Kendall’s tau *b* measure
				No	Possible	Definite	Value
T1	238	37	Research diagnosis of ASC	2 (5%)	13 (35%)	22 (60%)	0.60 (**)
			No research diagnosis of ASC	152 (76%)	46 (23%)	3 (1%)	
T2	180	27	Research diagnosis of ASC	2 (7.5%)	2 (7.5%)	23 (85%)	0.57(**)
			No research diagnosis of ASC	117 (77%)	22 (14%)	14 (9%)	
T3	80	12	Research diagnosis of ASC	1 (8%)	0 (0)	11 (92%)	0.49 (**)
			No research diagnosis of ASC	45 (66%)	11 (16%)	12 (17%)	
T4	30	3	Research diagnosis of ASC	0 (0)	1 (33%)	2 (67%)	0.33
			No research diagnosis of ASC	18 (67%)	0 (0)	9 (33%)	

*There were four children with a research diagnosis who had no practitioner data for any time point

**Correlation is significant at 0.001 level, two tailed

**Table 4:** Agreement between practitioner and research diagnosis across four time points with Kendall’s tau b coefficient score in the cases where the DAWBA was disclosed prior to practitioner assessment (**Correlation is significant at 0.001 level, two tailed)

Time point	Number of children with practitioner data with DAWBA disclosed (%)	Number of children with a research diagnosis of ASC with DAWBA disclosed (%)	Type of diagnosis	ASC according to practitioner *N *(%)			Kendall’s tau *b*
				No	Possible	Definite	
T1	117/238 (49%)	19/37 (51%)	Research diagnosis of ASC	2 (11%)	4 (21%)	13 (68%)	0.60 (**)
			No research diagnosis of ASC	76 (77%)	21 (22%)	1 (1%)	
T2	85/180 (47%)	13/27 (48%)	Research diagnosis of ASC	1 (8%)	0 (0)	12 (92%)	0.61(**)
			No research diagnosis of ASC	56 (78%)	11 (15%)	5 (7%)	
T3	43/80 (54%)	4/12 (33%)	Research diagnosis of ASC	0 (0)	0 (0)	4 (100%)	0.42
			No research diagnosis of ASC	23 (60%)	8 (20%)	8 (20%)	
T4	17/30 (57%)	1/3 (33%)	Research diagnosis of ASC	0 (0)	0 (0)	1 (100%)	0.39
			No research diagnosis of ASC	12 (75%)	0 (0)	4 (25%)

**Table 5:** Agreement between practitioner and research diagnosis across four time points with Kendall’s tau b coefficient score in the cases where the DAWBA was not disclosed prior to practitioner assessment (**Correlation is significant at 0.001 level, two tailed)

Time point	Number of children with practitioner data without DAWBA disclosed (%)	Number of children with a research diagnosis of ASC without DAWBA disclosed (%)	Type of diagnosis	ASC according to practitioner *N *(%)			Kendall’s tau b
				No	Possible	Definite	
T1	116/238 (49%)	16/37 (43%)	Research diagnosis of ASC	0 (0)	8 (50%)	8 (50%)	0.58 (**)
			No research diagnosis of ASC	74 (74%)	24 (24%)	2 (2%)	
T2	92/180 (51%)	13/27 (48%)	Research diagnosis of ASC	1 (8%)	2 (15%)	10 (77%)	0.52(**)
			No research diagnosis of ASC	59 (75%)	11 (14%)	9 (11%)	
T3	37/80 (46%)	8/12 (66%)	Research diagnosis of ASC	1 (12%)	0 (0)	7 (88%)	0.58(**)
			No research diagnosis of ASC	22 (76%)	3 (10%)	4 (14%)	
T4	13/30 (43%)	2/3 (67%)	Research diagnosis of ASC	0 (0)	1 (50%)	1 (50%)	0.22
			No research diagnosis of ASC	6 (55%)	0 (0)	5 (45%)	

### Agreement between referrer and research diagnosis

A total of 59 children (19.5%) were referred with a description in the correspondence that strongly suggested that ASC might be present. Of these, 29 (49%, Kappa=0.51, *p* = 0.00) were assigned a research diagnosis of ASC. This is shown in Table 2.

### Agreement between referrer and practitioner diagnosis

Of the 59 children referred with a suggestion of ASC, 19 (32%) were given a definite and 25 (42%) a possible practitioner ASC diagnosis. The Kendall’s tau b chance-corrected agreement between referrers and practitioner diagnosis at assessment was 0.36 (63%, *P* = 0.00).

## Agreement between practitioner and research diagnosis

Table 2 indicates the level of agreement between the DAWBA research diagnosis and practitioners at each time point, and indicates moderate initial levels of agreement between practitioners and the research diagnosis. Most children with a research diagnosis of ASC were reported to have a definite diagnosis of ASC by practitioners at some point. Similarly, few children with a research diagnosis of ASC were reported as definitely NOT having an ASC; most disagreements between practitioners and the research diagnoses were among children identified as having possible ASC by practitioners among the group without an ASC research diagnosis.

### Diagnostic stability

**Fig. 2:**
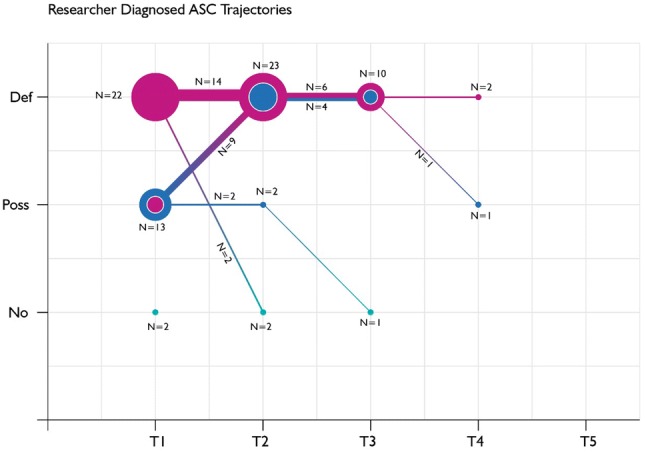
Diagnostic trajectory of children with a research diagnosis of ASC, according to practitioner diagnoses reported at each follow-up

**Fig. 3:**
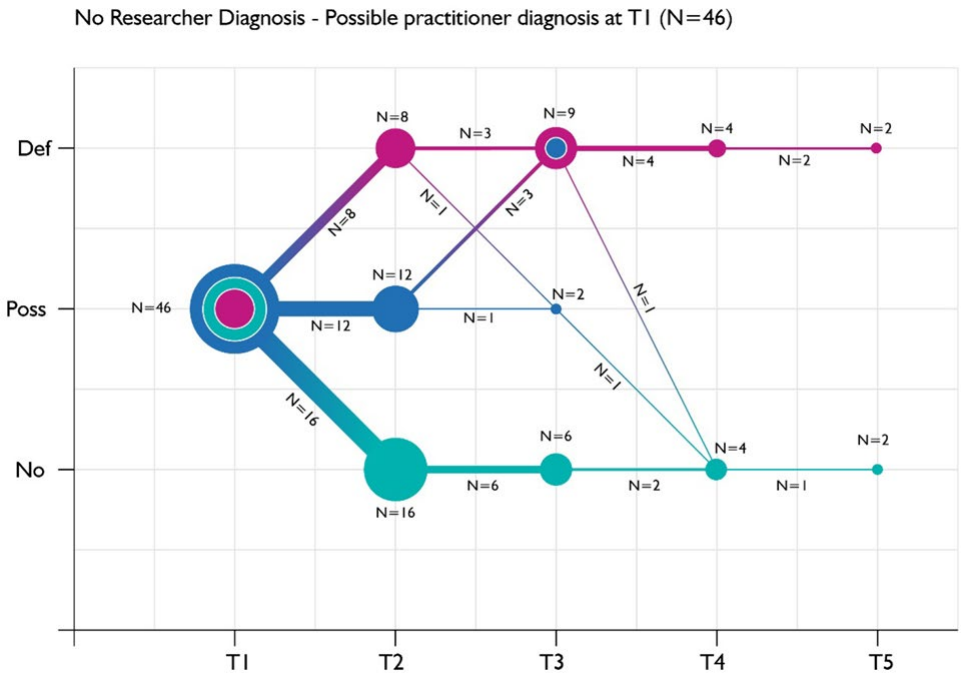
Diagnostic trajectory of children with no research diagnosis of ASC and a practitioner diagnosis of possible ASC at baseline, according to practitioner diagnoses reported at each follow-up

**Fig. 4:**
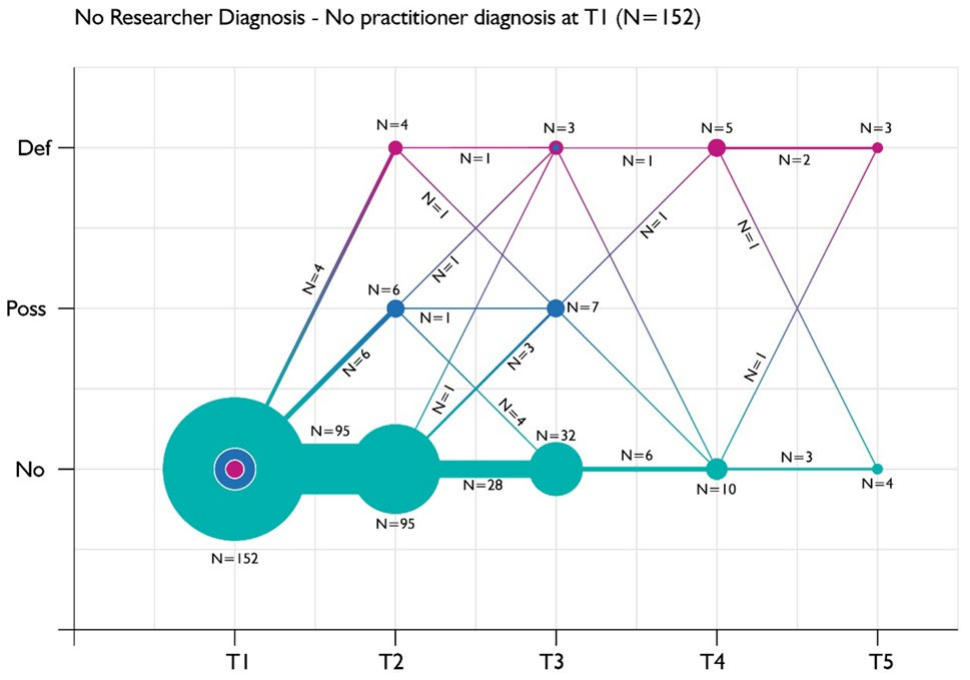
Diagnostic trajectory of children with neither a research diagnosis of ASC nor a practitioner diagnosis of ASC at baseline, according to practitioner diagnoses reported at each follow-up

As demonstrated in Table 3 and Figs. 2, 3, 4, there were high levels of attrition over time. Of those with a research diagnosis (Fig. 2), attrition was 27% from baseline to Time 2 and 70% to Time 3. Consequently, these figures are provided for information rather than analysis and any results must be interpreted cautiously.

Figure 2 describes the diagnostic trajectory of those children with a research diagnosis of ASC according to the practitioner diagnoses reported at each follow-up, and emphasises the strong level of agreement between the practitioners and the research diagnosis, as well as consistency in the practitioner diagnoses of ASC over time. The size of the circles at each time point reflects the number of children with that practitioner ASC diagnosis at that time point and the thickness of the line between time points reflects the number of children following that diagnostic trajectory. Of the 41 children with a research diagnosis of ASC, four had no practitioner data for any time point; therefore, they could not be included. The recognition of ASC, in terms of a “definite” practitioner diagnosis, occurred in 31/37 children (84%) with a clinically rated research diagnosis within  six months of CAMHS attendance (Kappa=0.57, *p* = 0.00): 22 (71%) of these cases were diagnosed as “definite” at baseline and the further 9 (29%) were diagnosed as “definite” at Time 2. There were no new practitioner diagnoses of ASC among the 37 children with a research diagnosis after Time 2, only shifts in certainty of diagnosis from possible to probable or vice versa.

Figure 3 illustrates the trajectory of practitioner diagnoses over time among children with no research diagnosis of ASC but with a baseline possible practitioner diagnosis over the five time points. There is considerable change between baseline and the first 6-month follow-up, with similar numbers of children moving from possible to definite and from possible to no ASC. There was less data on these children in later follow-ups, but that available suggested some individuals continue to experience marked fluctuations in diagnoses over time. Two children still had a possible ASC diagnosis after 1 year. Of the three children with a definite practitioner diagnosis of ASC at Time 1 who did not have a research diagnosis, one child was still reported to have a definite diagnosis at Time 2. Sadly, there was no further data from these children.

Twelve more children were diagnosed as “definite” at Time 2 (Fig. 3); for three children (25%), this was a new diagnosis. A further seven cases were diagnosed as “definite” at Time 3, with one (14%) of these a new diagnosis. A further three cases were diagnosed as “definite” at Time 4, all of which were new diagnoses. There was one new case diagnosed as “definite” at Time 5 or 2 years after initial assessment.

Similarly, Fig. 4 illustrates the trajectory of practitioner diagnoses over time among children with neither a research or practitioner diagnosis of ASC; as would be expected, most children were never reported to have ASC by practitioners, but a minority of children seemed to have marked fluctuations in practitioner reports of their diagnoses in relation to ASC over time.

### Severity

As Table 6 indicates, children with possible or no practitioner report of an ASC had significantly better prosocial skills (F(1,300)=41.25, *p* = 0.00) and fewer peer problems (F(1,300)=36.68, *p* = 0.00) compared to those with a research diagnosis of ASC; but they were also less impaired (F(1,300)=26.20, *p* = 0.00) (Table 3). There was no statistically significant difference in the SDQ total difficulties scores between those with or without a researcher diagnosed ASC (F(1,300)=3.82, *p* = 0.52), which is to be expected given that all these children were attending CAMHS and so would be expected to report significant levels of psychopathology and impact. Among children without a research diagnosis, children with no practitioner diagnosis had significantly lower mean peer problems (F(1,199)=7.34, *p* = 0.007) and greater mean prosocial skills (F(1,199)=3.94, *p* = 0.04) than those with possible or definite practitioner ASC diagnosis but again there was no significant difference between these two groups in their total difficulties or impact scores.

**Table 6:** Mean baseline scores from SDQ subscales, SDQ impact score and SDQ total difficulty scores according to the presence/absence of a research diagnosis of ASC and practitioner assessment at baseline

	SDQ prosocial subscale mean score (SD)	SDQ peer problem subscale mean score (SD)	SDQ total difficulties mean score (SD)	SDQ impact mean score (SD)
National norms in 5–10-year-olds (see www.sdqinfo.org)	8.6 (1.6)	1.4 (1.7)	8.6 (5.7)	0.3 (1.1)
Children with a researcher diagnosed ASC (*N* = 41)	4.76 (2.36)	5.63 (2.02)	22.24 (7.38)	6.80 (2.46)
Children without a researcher diagnosed ASC at baseline				
Children with a possible/definite practitioner diagnosis (*N* = 49*)	6.55 (1.91)	4.02 (2.23)	21.63 (6.39)	4.86 (2.49)
Children with no practitioner ASC diagnosis (*N* = 152)	7.28 (2.34)	3.03 (2.21)	19.63 (6.26)	4.44 (2.80)

*There were only three children with a definite practitioner diagnosis who did not have a research diagnosis, so the possible and definite diagnoses were combined

## Discussion

In this secondary analysis of data from a clinical cohort of primary school-aged children, we detected a moderate level of agreement between the clinically rated research diagnosis and the practitioner’s assessment of whether or not a child had an ASC on first attendance at the clinic, which was similar at Time 2 before decreasing at subsequent time points. Presenting problems indicated by referrers also showed fair agreement with research diagnoses. The initial levels of agreement are in keeping with previous studies, where fair to moderate levels of agreement have been found when comparing the DAWBA with a clinical diagnosis in teenagers [33] and in studies comparing other standardised diagnostic interviews and clinical evaluations [34]. Jensen and Weisz compared practitioner’s assessment to the diagnosis of the Diagnostic Interview Schedule for Children and Adolescents (DISC) in an outpatient setting [35]. Agreement was poor for all individual disorders (such as separation anxiety) and ranged from poor to fair for broader diagnostic clusters (such as anxiety disorders). Practitioners tended not to report comorbid disorders, which might have implications for children who have ASC who are often reported to have particularly high rates of comorbid conditions [36, 37]. In an extension to this work, Hawley and Weisz [38] compared the level of agreement between parents, therapists and children about the nature of the difficulties that intervention was addressing. They reported that in more than three-quarters of cases, treatment began without consensus among the triad, with the lowest level of agreement being between child and parent, and the highest between parent and therapist. A failure to agree on the nature of the problem might be expected to undermine attempts to intervene.

Weinstein and colleagues [39] compared diagnoses from the DISC with practitioner’s admission diagnoses and also reported very low levels of agreement; Kappas ranged from 0.03 to 0.17 (*M* = 0.10). A later study compared the same DISC diagnoses with the practitioner’s discharge diagnoses and found similarly poor agreement [40]; Kappas ranged from –0.07 to 0.22 (*M* = 0.11). This study was based on an inpatient sample, while the great majority of real-world child mental health assessment takes place in the community [41], which is a setting where diagnostic agreement may arguably be more difficult to obtain, given the briefer contact between staff and children. The poor levels of agreement in these studies do not necessarily mean that the research standardised assessments are correct and the practitioners are incorrect. Although not perfect, when compared to other indicators of validity, standardised assessments have greater validity and reliability than clinician-generated diagnoses in isolation, which is why research funding is rarely granted without the application of standardised assessment. For example, Basco et al. reported that standardised assessments were more likely than clinician diagnoses to agree with reference standard assessments generated by experts who reviewed all available information including medical records, clinician diagnoses and the results of the standardised assessment [42].

The decreasing level of agreement that we report over time could be due to imprecise estimates given the diminishing sample, or to shifts in some children’s difficulties since the DAWBA was completed only at baseline and children’s difficulties may change as they mature. However, national survey data from the UK suggest that there is also considerable stability in psychopathology over time with half the children meeting DSM IV criteria at baseline meeting diagnostic criteria for the same disorder three years later [43]. The national survey sadly included too few children with ASC to study homotypic persistence. We would not expect children to develop neurodevelopmental problems de novo, but comorbidity with other disorders that might develop and change over time is common and might complicate the presentation to CAMHS [37].

Most children with a research diagnosis of ASC were recognised within the first six months of attendance at CAMHS and most children without a research diagnosis of ASC were consistently reported not to have this condition by practitioners. This may reflect the type of cases that were picked up by the DAWBA, which have been suggested to be the more severe, clinically relevant cases [44]. Previous studies have reported that children with classical autism demonstrated greater diagnostic stability in relation to their difficulties over time than children who were diagnosed as having pervasive developmental disorder not otherwise specified or atypical autism [45, 46]. Such diagnostic instability within the different sub-types of ASC was demonstrated by Lord and colleagues [47], and underpins the move to a single category in the fifth edition of the Diagnostic and Statistical Manual [3]. Practitioners were not asked to report ASC in this level of detail and the small number of children with ASC diagnoses assigned by practitioner or research assessment prohibits such an analysis in the current study.

In our study, once assessed by CAMHS, most children with ASC receive a diagnosis within the first six months, which approximates to The National Autism Plan for Children recommendation that time from referral to diagnosis should not exceed 17 weeks [10]. It also runs counter to reports of long delays and multiple assessments reported by others [19–22, 24, 25]. We do not, however, have details on when these families first sought advice or which services they may have been in contact with prior to the index presentation to CAMHS.

Approximately one-third (32%) of children with a research diagnosis of ASC remained in the study 12 months after their initial assessment. Although we are unable to comment with certainty on the outcome of those lost to follow-up, some had been discharged from CAMHS. As a lifelong neurodevelopmental condition, one might expect a longer duration of treatment following such a diagnosis. A recent report of the Education Policy Institute's Mental Health Commission has highlighted the “single episode of care” model and condition-specific commissioning as problematic for children with neurodevelopmental difficulties in particular, potentially resulting in children with very high levels of need unable to access services due to not meeting specific criteria [48].

We would expect school age children with an ASC to have fewer prosocial skills and poorer peer relationships compared to their peers as parental SDQ subscales indicated, but parental reports suggest a similar pattern of difficulties, albeit less severe, among children whose practitioners assessed them to have a possible or definite ASC but who did not reach diagnostic criteria on the DAWBA. These less severe difficulties may contribute to diagnostic uncertainty and suggest subclinical levels of problems that might never-the-less contribute to impairment and to diagnostic confusion. As reported above, two trials of standardised assessment have been conducted to date [31, 33], which may not have been adequately powered to detect a difference in the detection of ASCs between the trial conditions (disclosure or not of the assessment to the clinical team). Our findings suggest that disagreement was mainly between the research diagnosis and practitioner reports of a possible ASC, with a remarkable level of fluctuation in practitioner-reported diagnoses over time. The potential confusion, frustration and delayed access may account for the negative referral experiences reported by others [13, 21, 22]. The application of standardised assessment measures might be particularly informative when there is diagnostic uncertainty or fluctuation, which could be tested empirically.

### Methodological considerations

This study benefitted from the use of a robustly validated multi-informant standardised diagnostic assessment and parental-reported measure of psychopathology. The diagnoses were clinically rated by a senior child psychiatrist (TF) with extensive experience of this measure in national surveys [36, 49]. However, practitioner report matched DAWBA diagnoses, but was not a validated measure. The study recruited from consecutive referrals, and while the response rate was relatively low, analysis of respondents and non-respondents suggested that differences in background characteristics related to inclusion and exclusion criteria [26]. As with all secondary analyses, we were constrained by the data available, and more detail about children’s difficulties and other services used would be informative. Similarly, we lacked information about how diagnoses were made, what was communicated about it to the families and how assessments fed into intervention. The fairly small sample size, reduced by the focus on children with ASCs, limited the power available for the analysis of background characteristics that might be useful for practitioners in increasing the accuracy and speed of assessment. Furthermore, the high rate of attrition is a potential source of bias. While some children may have been discharged from CAMHS and no longer require any support, others may have ongoing difficulties which are not being addressed. We are unable to comment on the diagnoses and outcomes of those lost to follow-up.

## Conclusions

Child health mapping suggests that one in every ten children utilising CAMHS has an ASC [50]. Our findings suggest that where practitioners are confident that a child definitely does or does not have an ASC, there was considerable agreement between practitioner and research diagnoses and clinical diagnoses were stable over time. However, for some children, initial diagnostic uncertainty led to confusing and prolonged fluctuations in practitioner assessments that may have undermined both engagement and intervention. The use of standardised assessments and observations might be particularly helpful for these children and could be evaluated further.
